# Immunolabelling of human metaphase chromosomes reveals the same banded distribution of histone H3 isoforms methylated at lysine 4 in primary lymphocytes and cultured cell lines

**DOI:** 10.1186/s12863-015-0200-5

**Published:** 2015-04-29

**Authors:** Edith Terrenoire, John A Halsall, Bryan M Turner

**Affiliations:** School of Cancer Sciences, College of Medical and Dental Sciences, University of Birmingham, Edgbaston, Birmingham, B15 2TT UK; West Midlands Regional Genetics Laboratory, Birmingham Women’s NHS Foundation Trust, Mindelsohn Way, Edgbaston, Birmingham, B15 2TG UK; Present address : Service de Génétique, CHU de Tours, 2 Boulevard Tonnellé, 37044 Tours, Cedex 09, France

**Keywords:** Immunolabelling, Metaphase chromosome, Histone methylation, Human epigenome

## Abstract

**Background:**

Using metaphase spreads from human lymphoblastoid cell lines, we previously showed how immunofluorescence microscopy could define the distribution of histone modifications across metaphase chromosomes. We showed that different histone modifications gave consistent and clearly defined immunofluorescent banding patterns. However, it was not clear to what extent these higher level distributions were influenced by long-term growth in culture, or by the specific functional associations of individual histone modifications.

**Results:**

Metaphase chromosome spreads from human lymphocytes stimulated to grow in short-term culture, were immunostained with antibodies to histone H3 mono- or tri-methylated at lysine 4 (H3K4me1, H3K4me3). Chromosomes were identified on the basis of morphology and reverse DAPI (rDAPI) banding. Both antisera gave the same distinctive immunofluorescent staining pattern, with unstained heterochromatic regions and a banded distribution along the chromosome arms. Karyotypes were prepared, showing the reproducibility of banding between sister chromatids, homologue pairs and from one metaphase spread to another. At the light microscope level, we detect no difference between the banding patterns along chromosomes from primary lymphocytes and lymphoblastoid cell lines adapted to long-term growth in culture.

**Conclusions:**

The distribution of H3K4me3 is the same across metaphase chromosomes from human primary lymphocytes and LCL, showing that higher level distribution is not altered by immortalization or long-term culture. The two modifications H3K4me1 (enriched in gene enhancer regions) and H3K4me3 (enriched in gene promoter regions) show the same distributions across human metaphase chromosomes, showing that functional differences do not necessarily cause modifications to differ in their higher-level distributions.

**Electronic supplementary material:**

The online version of this article (doi:10.1186/s12863-015-0200-5) contains supplementary material, which is available to authorized users.

## Background

Our previously published work described how immunofluorescence microscopy could be used to provide an overview of the distribution of histone modifications across human metaphase chromosomes. Using metaphase chromosome spreads from lymphoblastoid cell lines (LCL) of normal karyotype and antisera to some key histone modifications, we showed that different histone modifications gave consistent and clearly defined banding patterns [1]. Various modifications linked to transcriptional activity, such as histone H3 tri-methylated at lysine 4 (H3K4me3), H3 acetylated at lysine 27 (H3K27ac) and H3 acetylated at lysine 9 (H3K9ac), gave the same staining patterns, with strongly staining regions distributed across the euchromatic chromosome arms. In contrast, the banding pattern was strikingly different for modifications associated with gene silencing such as H3 tri-methylated at lysine 27 (H3K27me3), which gave broad bands that often overlapped, but were not coincident with, the sharp bands containing modifications associated with transcriptionally active chromatin. H4 tri-methylated at lysine 20 (H4K20me3), a modification associated with heterochromatin formation [2], was largely centromeric [1]. We found that the distribution of active modifications was closely related to the distribution of regions rich in genes, CpG Islands (CGI) and SINE elements [1]. However, it is not clear to what extent the higher level distributions revealed by indirect immunofluorescence (IIF) microscopy reflect the specific functional associations of individual histone modifications, or how they are influenced by the differentiation status of the host cell, or by long-term growth in culture. Here, we address these issues by (i) defining the distribution of H3K4me3 across metaphase chromosomes from primary human lymphocytes stimulated to grow in short-term culture, and comparing this with our previous results in LCL, and (ii) comparing the distributions of H3K4me3, a modification associated with promoter regions [3,4] with that of H3K4me1, a modification also linked to transcriptionally active chromatin, but now known to be associated with enhancer regions [3,5].

## Results

### Distribution of H3K4me3 in chromosomes from primary lymphocytes and comparison with LCL

**Figure 1:**
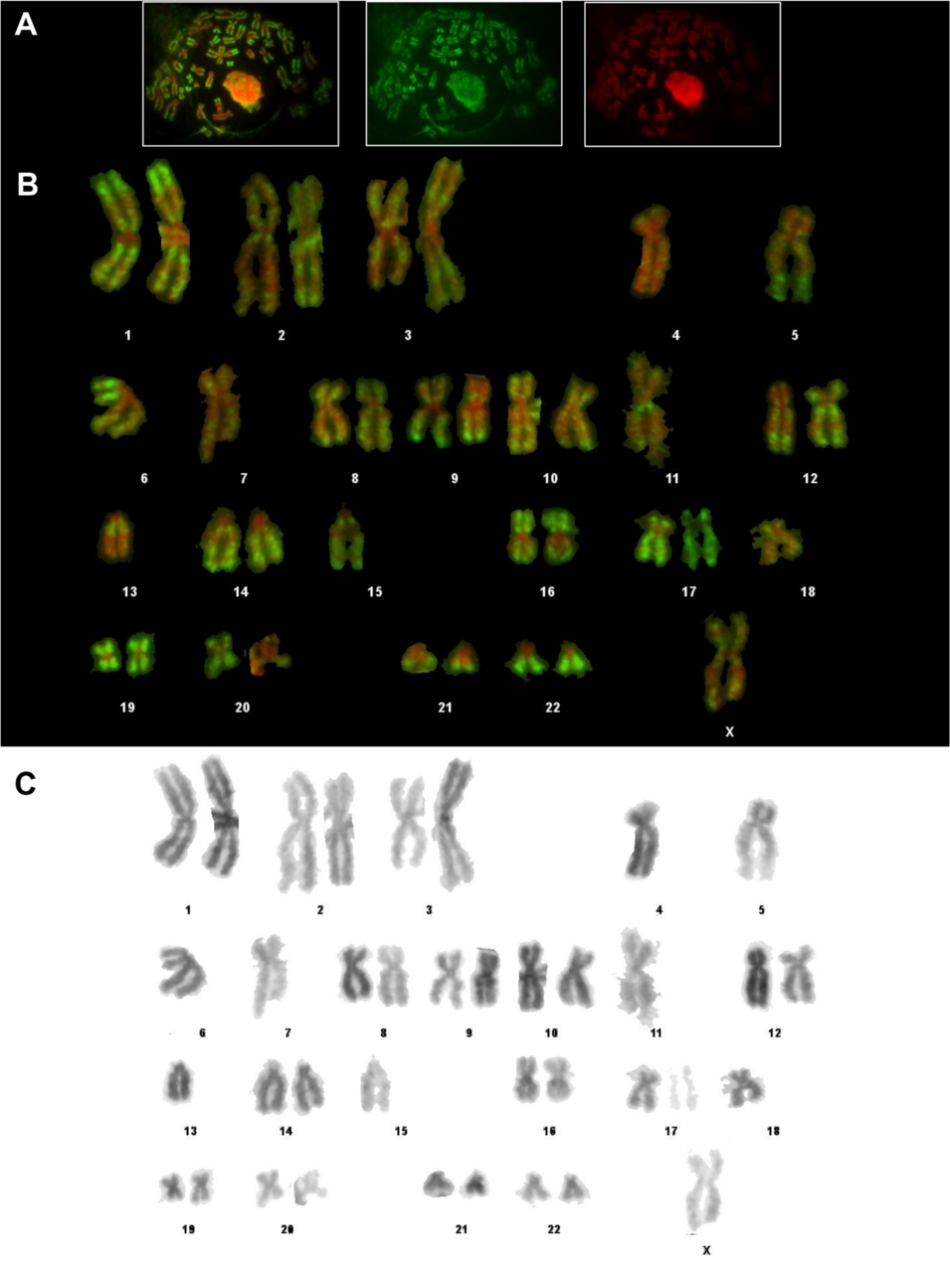
Immunostaining of H3K4me3 in metaphase chromosomes from human primary lymphocytes. **A**. Metaphase chromosome spread immunostained with rabbit antibody to H3K4me3 and fluorescein isothiocyanate (FITC) conjugated goat anti-rabbit (green stain); DNA was visualized with 4′,6-diamidino-2-phenylindole (DAPI, pseudocoloured red). The three panels show FITC + DAPI (left), FITC (centre) and DAPI (right). **B**. Immunostained karyotype constructed from the metaphase spread shown. **C**. Reverse DAPI (rDAPI) karyotype constructed from the same spread, shown in black to resemble conventional G-banding.

Metaphase chromosome spreads from cultured lymphocytes were immunostained with antibody to H3K4me3. A representative metaphase spread and karyotype are shown in Figures 1A and B respectively. Chromosomes were identified by size, centromeric index and reverse DAPI staining (Figure 1C). Centromeric heterochromatin (prominent at 1q12, 9q12 and 16q11.2) lacks antibody staining (FITC, green), showing up as bright red (DAPI DNA counterstain, pseudo-coloured red). The most gene-rich human chromosome 19, is uniformly and consistently brightly stained, while the similarly sized but gene-poor chromosome 18 is pale-staining overall (Figure 1B). The rest of the genome shows a pattern of brightly and weakly stained regions. Known gene-rich regions such as 1p32-pter, 6p21, 9q34-qter, all show strong staining for H3K4me3, as described previously for LCL [1].

**Figure 2:**
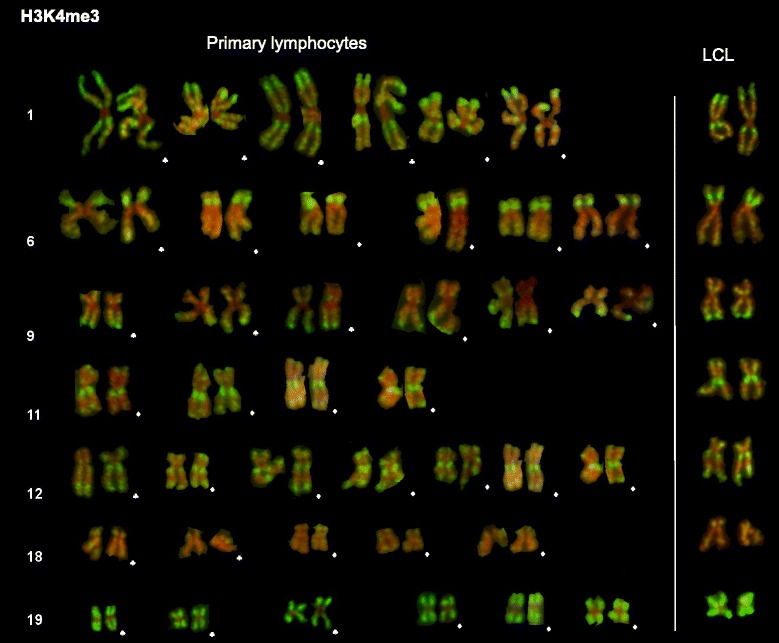
Selected chromosomes from human primary lymphocytes and lymphoblastoid cells immunostained for H3K4me3. Selection of chromosomes 1, 6, 9, 11, 12 , 18 and 19 from human primary lymphocytes is presented to allow the consistency of banding between metaphase spreads and individuals to be examined. (♣) donor 1, (♦) donor 2. Typical examples of each immunostained chromosome from human lymphoblastoid cells (LCL) are shown on the right.

Although the fragility of unfixed chromosomes from primary lymphocytes made it difficult to prepare, with confidence, complete karyotypes from individual metaphase spreads, pairs of homologous chromosomes were readily identifiable. The karyotype in Figure 2 shows pairs of seven chromosomes with characteristic and well-defined banding patterns (1, 6, 9, 11, 12, 18 and 19) from metaphase spreads from each of two donors. (The ten original spreads are shown in Additional file 1). Banding is consistent between sister chromatids, (particularly visible on chromosomes 1, 6, 9, 11 and 12), and from one homologue pair to another (Figure 2). It is noteworthy that the overall pattern of immunofluorescent bands is maintained even when homologues have been differentially stretched or distorted (eg. Figure 1B, chromosome 3). Heritable differences in chromatin compaction between metaphase chromosome homologues have been detected by differences in accessibility of specific DNA probes [6]. Such differences may be detectable by immunostaining, but will require higher resolution analysis and a larger number of donors than used for the present study. Overall, the major regions rich in H3K4me3 on metaphase chromosomes from primary human lymphocytes are indistinguishable from those previously described on chromosomes from LCL [1] and shown on the extreme right of each row of homologues (Figure 2, see also Additional file 1).

### Comparison of the distribution of H3K4me3 and H3K4me1 in primary lymphocytes

**Figure 3:**
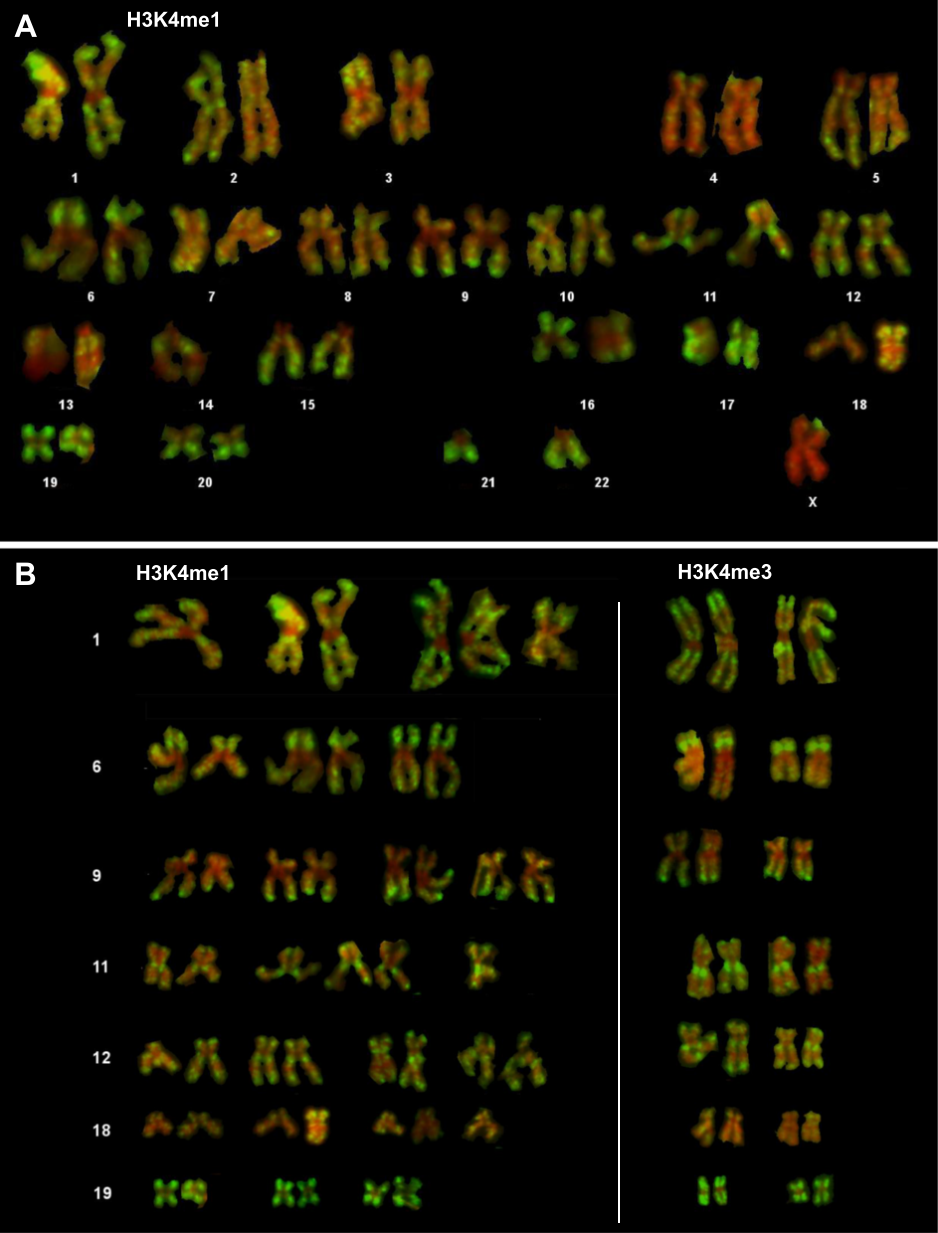
Selected chromosomes from human primary lymphocytes immunostained for H3K4me1 or H3K4me3. **A**. Karyotype constructed from metaphase spread immunostained with antibodies to H3K4me1. Where only a single chromosome is shown (14, 21, 22, X), the homologue could not be reliably identified. The metaphase spread is from a female donor and the weakly stained X shown is likely to be the inactive X. **B**. Selection of chromosomes 1, 6, 9, 11, 12, 18 and 19 to allow examinination of the consistency of H3K4me1 banding between metaphase spreads and to allow comparison with H3K4me3 banding (right hand side). All chromosomes shown were from donor 1.

To explore the extent to which the distribution of histone modifications along metaphase chromosomes is dependent on the functional associations of the modification, we analyzed the banding pattern obtained with antisera to H3K4me1, a modification associated with gene enhancer regions [3,5]. A karyotype is shown in Figure 3A. There is a clear immunofluorescent banding pattern, with centric heterochromatin remaining unstained, strong staining of gene-rich regions (eg. 1p32-pter, 6p21, 9q34-qter, 11q13, 11q23-qter, 12p13, 12q13, 12q24 and chromosome 19) and weak staining of known gene-poor regions such as chromosome 18 (Figure 3A). The composite karyotype in Figure 3B shows the consistency of H3K4me1 staining on chromosomes from different metaphase spreads, and allows direct comparison with H3K4me3 staining. The major bands revealed by the two antibodies are indistinguishable, showing that, at this level, the two modifications are coincident, despite their different functional associations.

## Discussion

By immunofluorescence microscopy, different histone modifications show distinctive distributions across human metaphase chromosomes; H3K20me3 is associated primarily with centric heterochromatin [1,7], while H3K27me3, a modification closely linked to gene silencing through the Polycomb complex [8], is distributed as broad bands, sometimes incorporating gene-rich regions but not restricted to such regions [1], finally H3K4me1, H3K4me3, H3K9ac and H3K27ac are all associated with regions rich in genes, CGI and SINE elements (present results and [1]). H4 acetylation gives banding that corresponds to the more sharply defined H3K4me3 bands [1] and in early experiments, was associated with gene-rich T-bands [9]. The explanation for these distinctive, high level banded distributions probably lies in the general functions with which the modifications are linked. H4K20me3 is required for chromatin condensation and heterochromatin compaction [7]. The multiple modifications that highlight gene-rich regions are all involved, in one way or another, in transcriptional activation, and their overall enrichment in gene-rich regions, irrespective of their exact functional involvements, is understandable. Epigenomic analyses [5] show that H3K4me1 and H3K4me3 are differently distributed at the gene level and below, but their distribution is indistinguishable at the 1-10Mb level revealed by chromosome immunofluorescent banding. Polycomb-associated modification H3K27me3 is well known to spread over wide genomic regions [8], and a role in suppressing extra-genic transcription would explain why its immunostaining reveals bands extending beyond gene-rich regions.

It remains uncertain whether the patterns of histone modification that define individual chromosome bands are a simple reflection of gene richness and/or ongoing transcription, or whether they play a determining role in chromatin packaging and intra-nuclear location at the Mb level. In this respect, it is of interest that the metaphase chromosome bands for H3K4me3 are indistinguishable between primary lymphocytes and lymphoblastoid cell lines. The lymphocyte metaphase spreads shown here are derived from short term culture and are likely to be from the first mitosis of these naturally post-mitotic cells. Our results show that banding is not noticeably influenced by the major epigenetic changes that must accompany establishment of lymphoblastoid cell line and adaptation to long-term growth in culture. It may be that at the highest level, the broad distribution of histone modifications (ie. banding) is determined by the need to adopt a specific, compacted chromosome structure at metaphase, and to maintain an established pattern of gene expression through mitosis, rather than the differentiation or growth status of the cell.

## Conclusions

At the light microscope level, the banded distribution across human metaphase chromosomes of two modified histones associated with active chromatin, H3K4me1 and H3K4me3, is the same, even though they are enriched at enhancers and promoters respectively and play different roles in transcriptional regulation.

The epigenetic changes that accompany adaptation to long-term growth in culture do not alter the banded distribution of H3K4me3 across human metaphase chromosomes.

## Methods

Peripheral blood was taken by venepuncture from healthy adult volunteers, with informed consent and ethical approval (National Research Ethics Committee, approval number Leeds East 07/Q1206/25). Mononuclear cells (PBMC) from 10ml aliquots of whole blood were isolated by LymphoPrep™ (Axis-shield). The white cell layer was aspirated, diluted to 50ml in PBS, spun down and washed twice in PBS and once in RPMI 1640 culture medium. Isolated PBMC were cultured and co-stimulated with PHA (5μg/ml ) and human interleukin-2 (30U/ml, both from Gibco ®) in RPMI1640 medium supplemented with 10% foetal bovine serum (Gibco) and 1% (v/v from Gibco stock solutions) L-glutamine and penicillin/streptomycin [1]. After 24 hours, cells were treated with colcemid (0.05μg/ml, Biochrom, Berlin) overnight (16h), prior to being spun down, washed twice in ice cold PBS, swollen in 75mM KCl (10min, at room temperature 1x10^5^ cells/ml) and spun onto glass slides using a Shandon Cytospin 4 (Thermo Electron corporation) [1]. Unfixed chromosomes from primary lymphocytes proved to be more fragile than those from LCL and to mitigate this, solutions were kept ice-cold and centrifugation was reduced to 1,200 rpm (Shandon cytospin 4, Thermo Fisher) for 5 min.

Immunostaining of metaphase spreads from native unfixed chromosomes was carried out exactly as described previously [1] using rabbit antisera to H3K4me1 (R204) and H3K4me3 (R612) and fluorescein isothiocyanate (FITC) conjugated goat anti-rabbit immunoglobulin (Sigma F1262) diluted x1000. Antisera were diluted in KCM buffer (120mM KCl, 20mM NaCl,10mM Tris/HCl pH 8.0, 0.5mM EDTA, 0.1% (v/v) Triton X-100) supplemented with 1% BSA (Sigma-Aldrich). Rabbit antisera were prepared -in-house and their specificities validated as previously described [1,10]. To stabilise labelled chromosomes, slides were fixed in 4% (v/v) formaldehyde in KCM buffer, before mounting in Vectorshield (Vector Lab, Peterborough, UK) supplemented with the DNA counterstain diamidino-2-phenylindole dihydrochloride (DAPI, Sigma) at 2 μg/ml, all as described [1]. Metaphase spreads were visualized on a Zeiss Axioplan 2 epifluorescence microscope under a x100 oil immersion lens. Metaphases chromosome capture and karyotyping were carried out with Smart Capture and Smart Type software (Digital Scientific, Cambridge, UK).

## Additional file

Additional file 1:
**Shows 10 separate karyotypes based on immunostaining metaphase chromosome spreads with antibodies to H3K4me3.**
