# Supplementary material for: Immunolabelling of human metaphase chromosomes reveals the same banded distribution of histone H3 isoforms methylated at lysine 4 in primary lymphocytes and cultured cell lines
Source: BMC Genet. 2015 Apr 29;16:44. doi: 10.1186/s12863-015-0200-5 (PMC4417270; doi:10.1186/s12863-015-0200-5)
Supplement: Additional file 1: — Shows 10 separate karyotypes based on immunostaining metaphase chromosome spreads with antibodies to H3K4me3. [file 12863_2015_200_MOESM1_ESM.pdf]

1

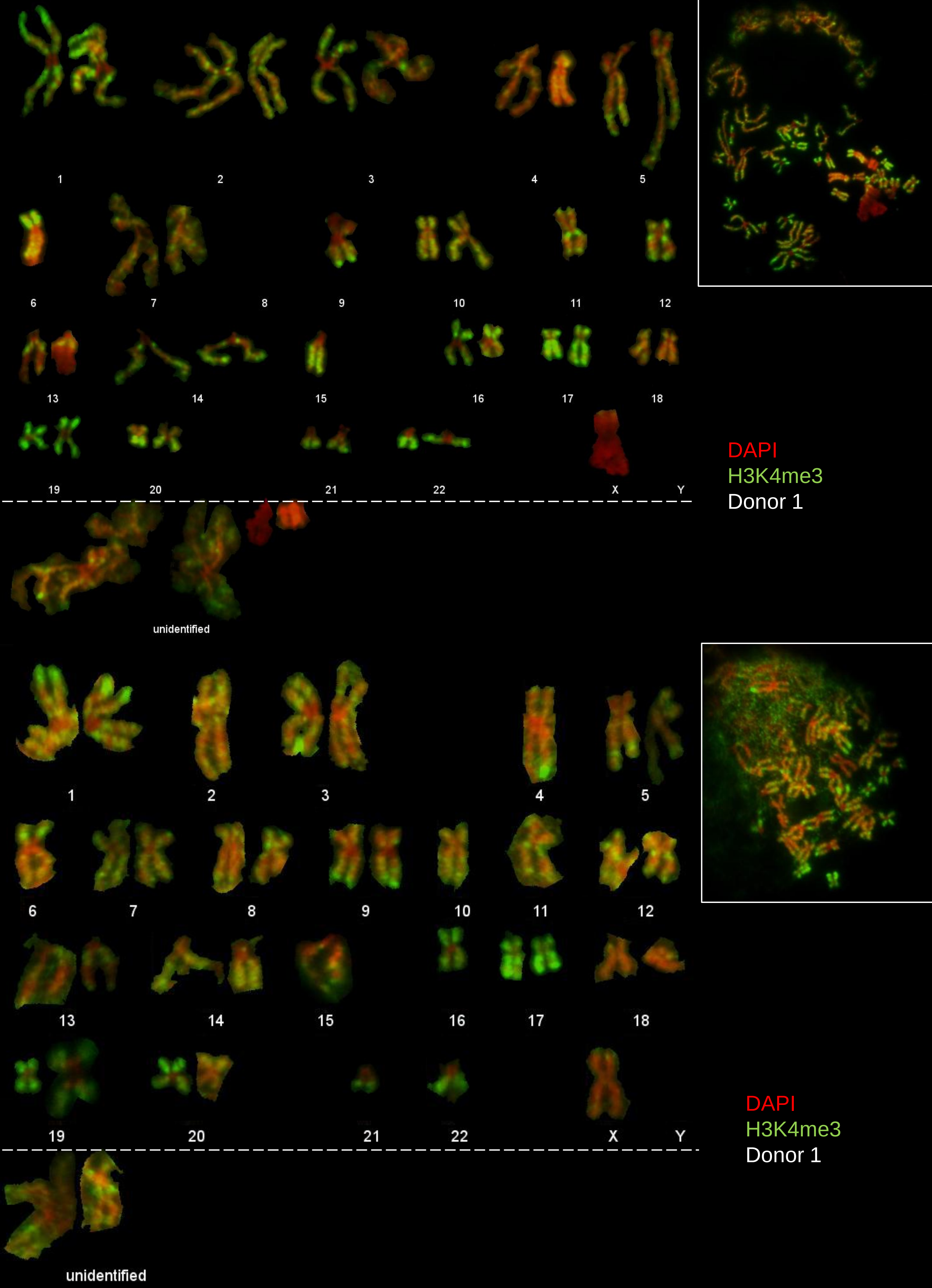
1 2 3 4 5
6 7 8 9 10 11 12
13 14 15 16 17 18
19 20 21 22 X Y
unidentified
DAPI
H3K4me3
Donor 1
1 2 3 4 5
6 7 8 9 10 11 12
13 14 15 16 17 18
19 20 21 22 X Y
unidentified
DAPI
H3K4me3
Donor 1

2

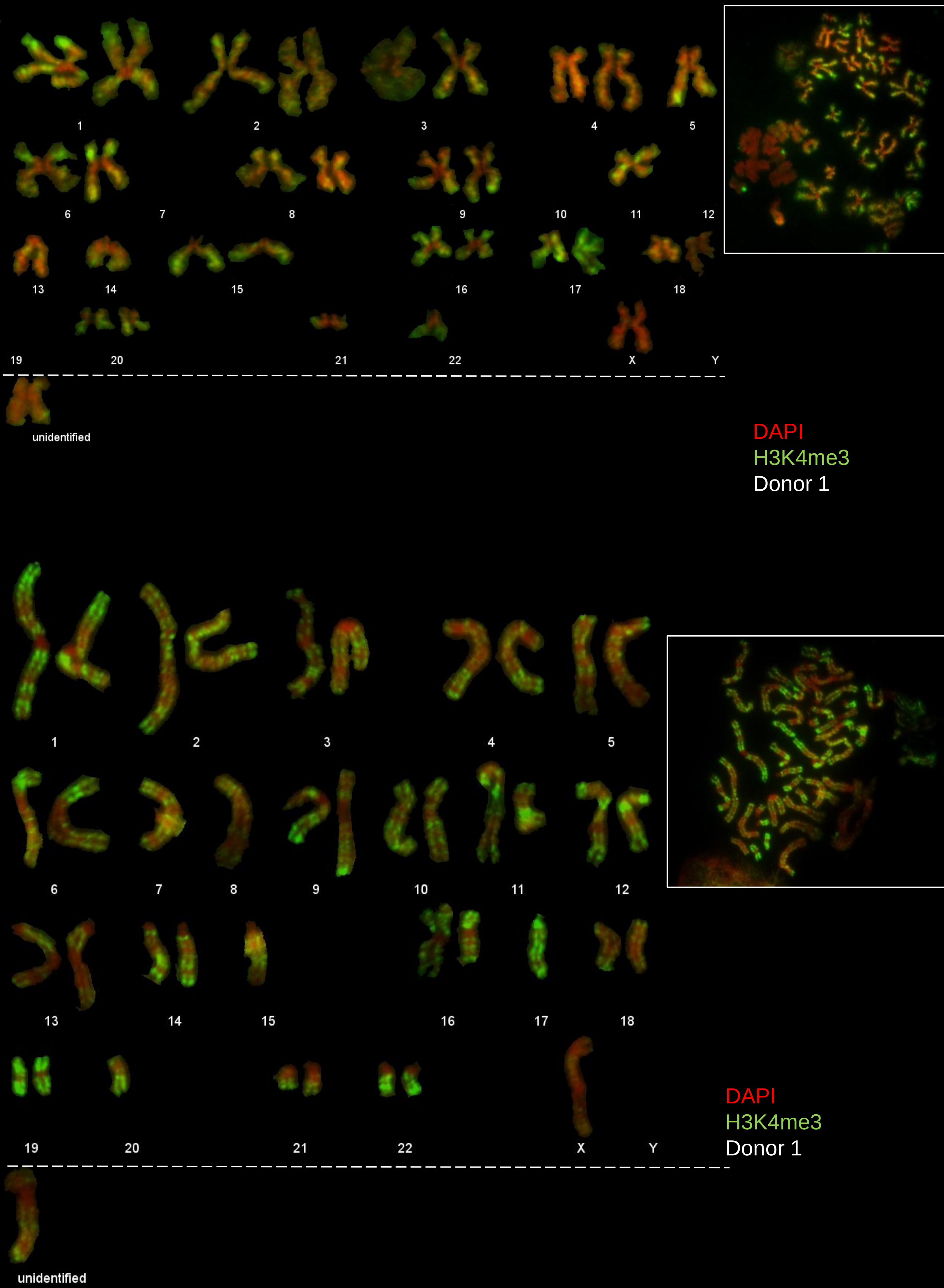
1 2 3 4 5
6 7 8 9 10 11 12
13 14 15 16 17 18
19 20 21 22 X Y
unidentified
DAPI
H3K4me3
Donor 1
1 2 3 4 5
6 7 8 9 10 11 12
13 14 15 16 17 18
19 20 21 22 X Y
DAPI
H3K4me3
Donor 1
unidentified

3

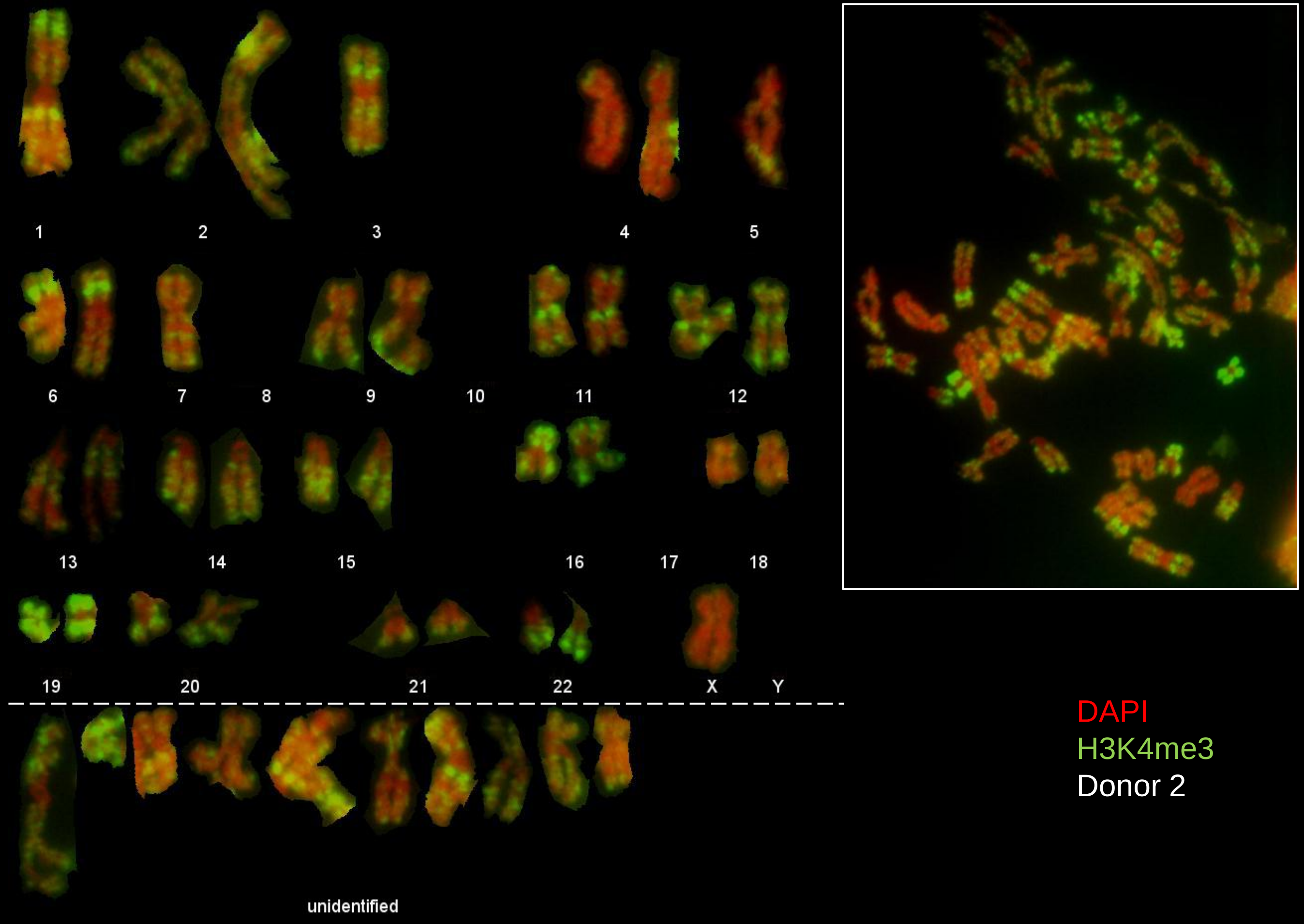
1
2
3
4
5
6
7
8
9
10
11
12
13
14
15
16
17
18
19
20
21
22
X
Y
unidentified
DAPI
H3K4me3
Donor 2

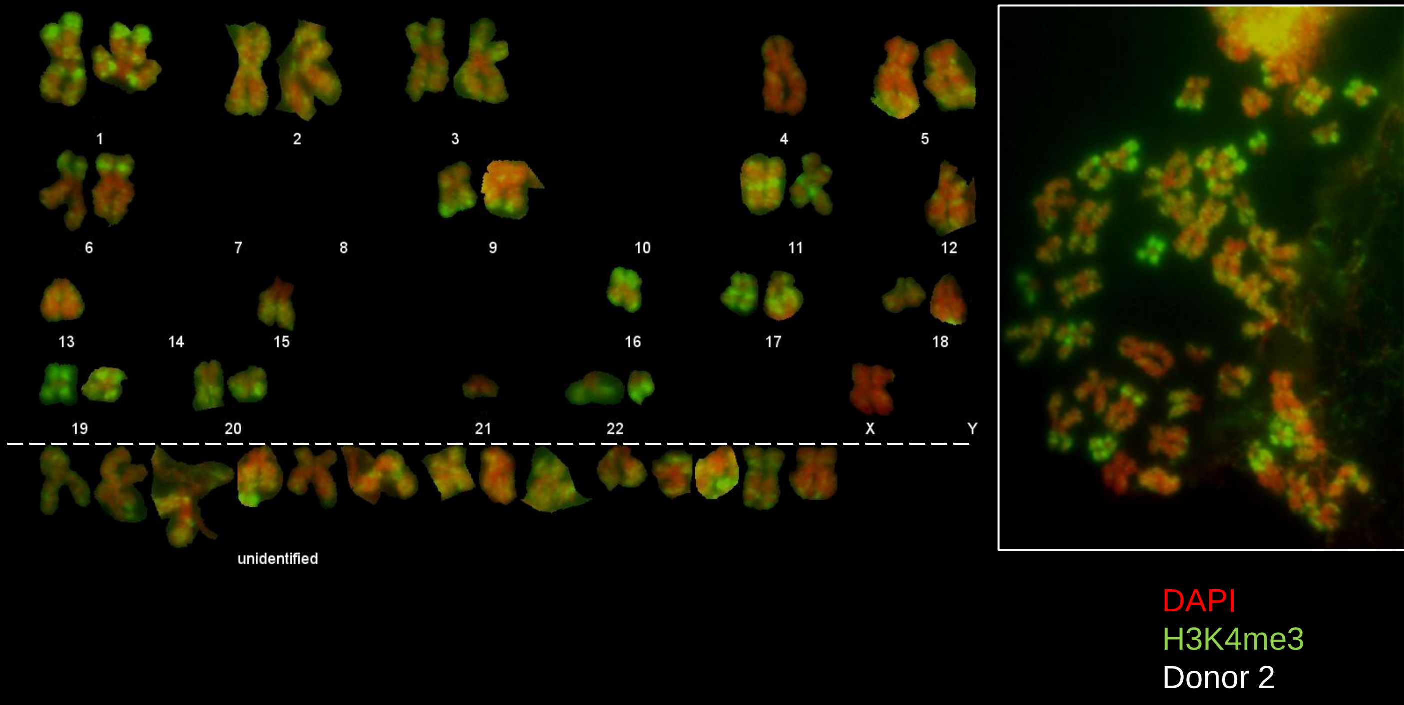
1
2
3
4
5
6
7
8
9
10
11
12
13
14
15
16
17
18
19
20
21
22
X
Y
unidentified
DAPI
H3K4me3
Donor 2

4

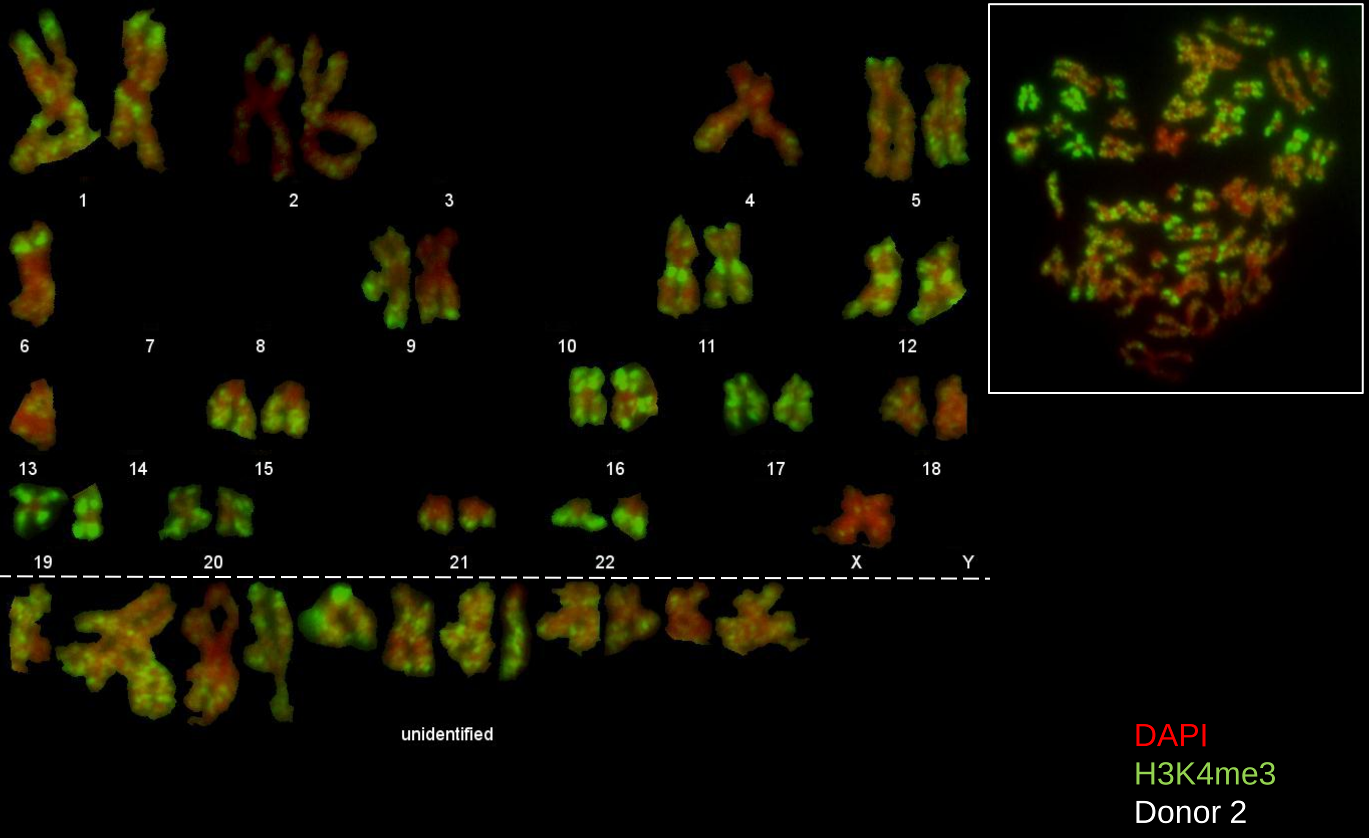
1
2
3
4
5
6
7
8
9
10
11
12
13
14
15
16
17
18
19
20
21
22
X
Y
unidentified
DAPI
H3K4me3
Donor 2

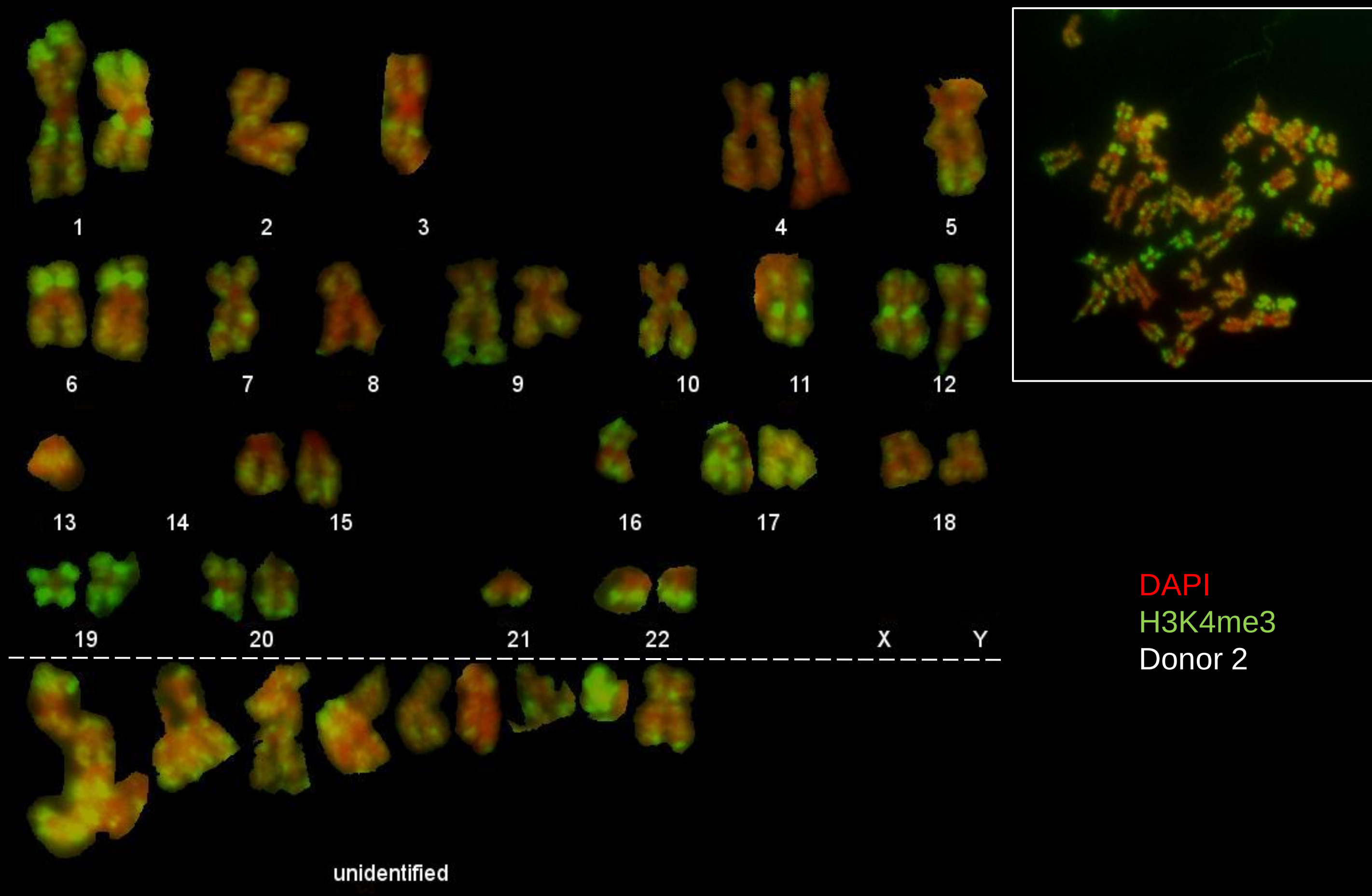
1
2
3
4
5
6
7
8
9
10
11
12
13
14
15
16
17
18
19
20
21
22
X
Y
unidentified
DAPI
H3K4me3
Donor 2

5

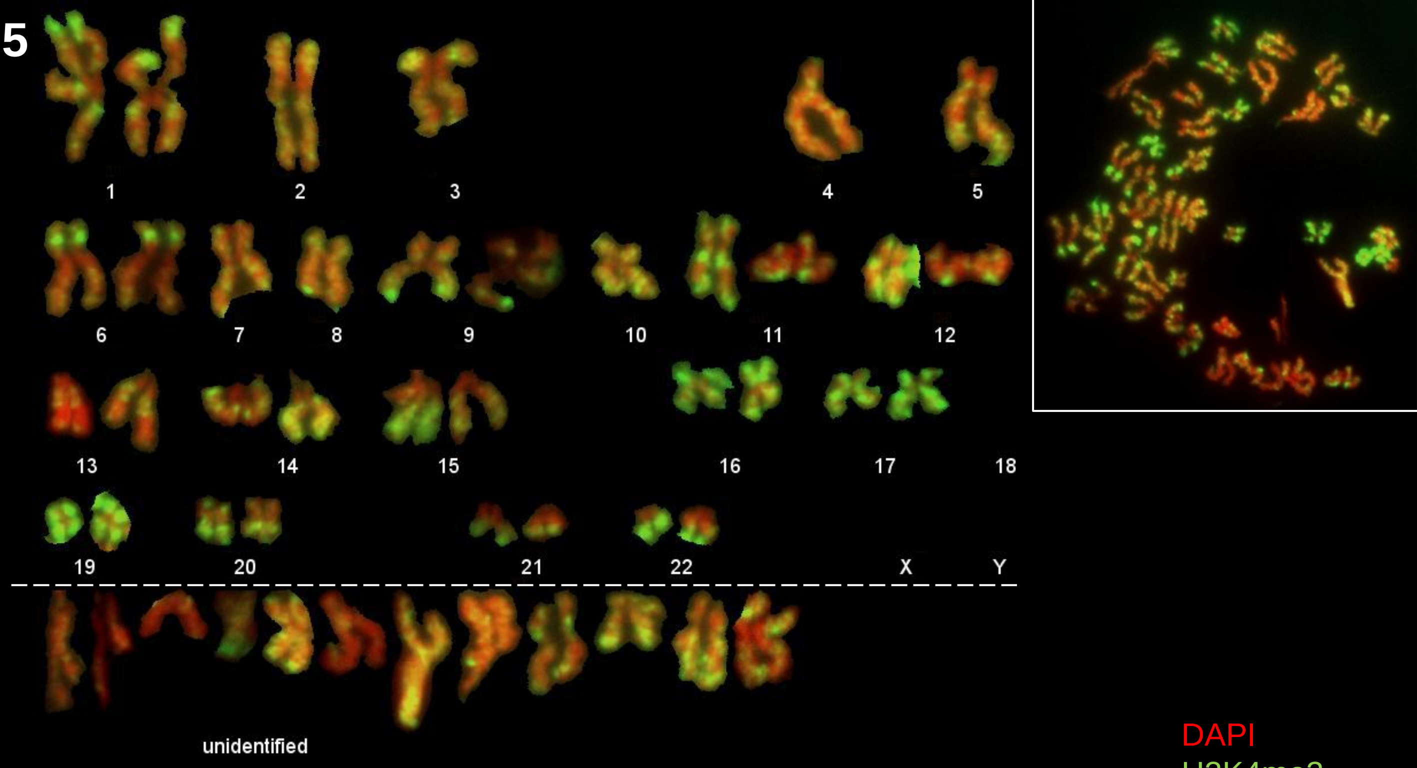
1
2
3
4
5
6
7
8
9
10
11
12
13
14
15
16
17
18
19
20
21
22
X
Y
unidentified
DAPI
H3K4me3
Donor 2

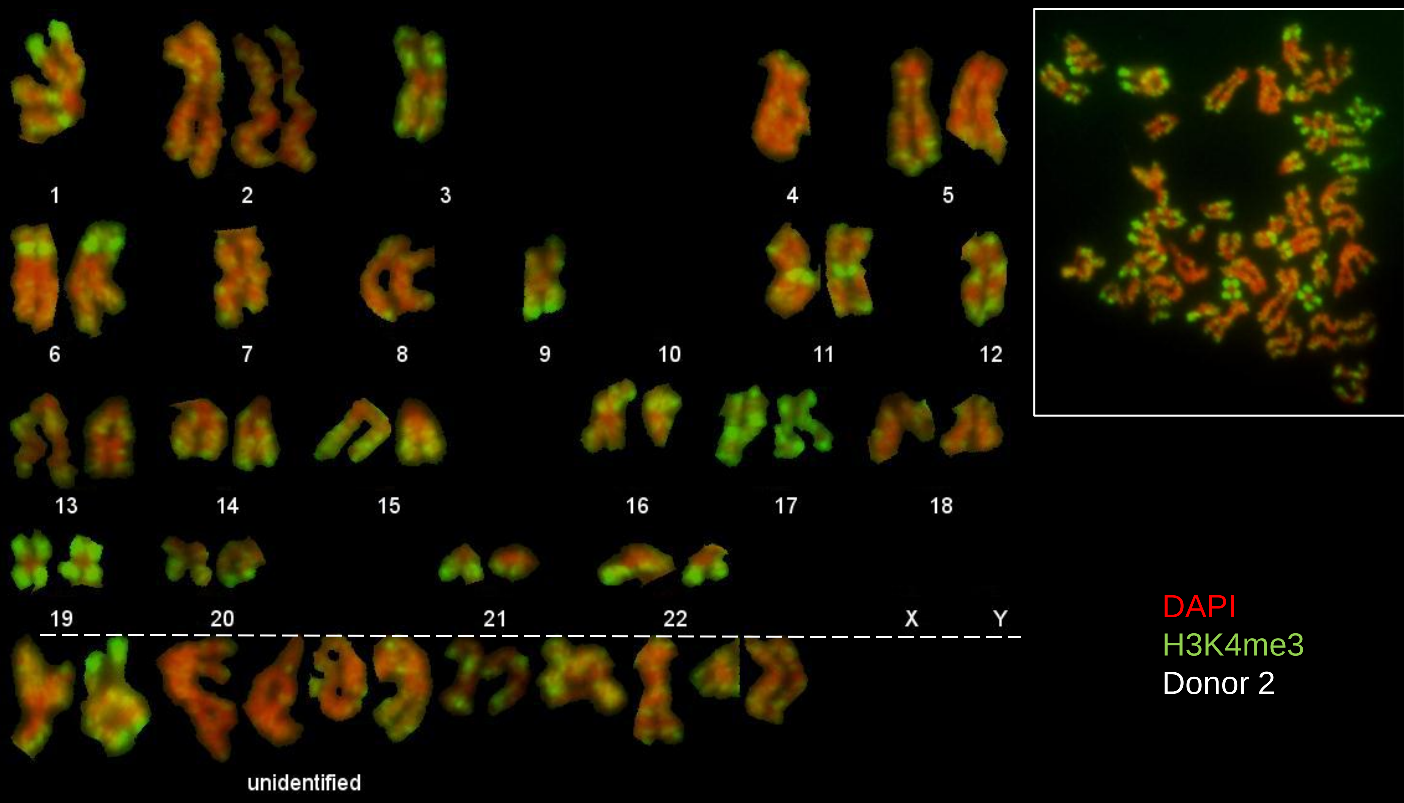
1
2
3
4
5
6
7
8
9
10
11
12
13
14
15
16
17
18
19
20
21
22
X
Y
unidentified
DAPI
H3K4me3
Donor 2
